# An efficient error correction algorithm using FM-index

**DOI:** 10.1186/s12859-017-1940-1

**Published:** 2017-11-28

**Authors:** Yao-Ting Huang, Yu-Wen Huang

**Affiliations:** Department of Computer Science and Information Engineering, National Chuang Cheng University, Chiayi, Taiwan

**Keywords:** FM-index, Next-generation sequencing

## Abstract

**Background:**

High-throughput sequencing offers higher throughput and lower cost for sequencing a genome. However, sequencing errors, including mismatches and indels, may be produced during sequencing. Because, errors may reduce the accuracy of subsequent de novo assembly, error correction is necessary prior to assembly. However, existing correction methods still face trade-offs among correction power, accuracy, and speed.

**Results:**

We develop a novel overlap-based error correction algorithm using FM-index (called FMOE). FMOE first identifies overlapping reads by aligning a query read simultaneously against multiple reads compressed by FM-index. Subsequently, sequencing errors are corrected by *k*-mer voting from overlapping reads only. The experimental results indicate that FMOE has highest correction power with comparable accuracy and speed. Our algorithm performs better in long-read than short-read datasets when compared with others. The assembly results indicated different algorithms has its own strength and weakness, whereas FMOE is good for long or good-quality reads.

**Conclusions:**

FMOE is freely available at https://github.com/ythuang0522/FMOC.

**Electronic supplementary material:**

The online version of this article (doi:10.1186/s12859-017-1940-1) contains supplementary material, which is available to authorized users.

## Background

High-throughput sequencing technologies have been widely used in the past decade for studying disease associations or deciphering genomes. The reads generated by next generation sequencing platforms (e.g., Illumina, Roche 454) may contain several types of errors including mismatches, insertions and deletions (collectively termed indels) [1]. These errors bring great challenges of subsequent genome assembly algorithms, because false read overlaps may be produced, which further leads to fragmented assembly or even misassembly. Furthermore, these errors will also increase the size of assembly graph due to erroneous vertices and edges, which implies requirement of larger memory usage and computational time. Therefore, prior to assembly, these reads are usually corrected in the hope of producing better assembled genome [2, 3].

In order to correct these errors, existing methods mainly rely on sufficient sequencing coverage for replacing less-frequent errors with more-frequent base. These algorithms can be roughly classified into the following three categories: (1) the *k*-mer frequency spectrum methods slide a fixed-sized *k*-mer window along the entire read and replace the low-frequent *k*-mers with high-frequent ones (e.g., QuorUm, Lighter, BLESS, Blue, Musket, and Quake) [4–9]; (2) Methods based on suffix tree (or array) are simialr to *k*-mer spectrum methods but different sizes of *k*-mers can be used adaptively in the suffix tree/array (e.g., Fiona, SHREC, HSHREC, HiTEC) [10–13]; (3) The overlap-based correction approaches first identify reads overlapping with the query (i.e., read to be corrected) via multiple-sequence alignment (MSA). Subsequently, errors are corrected according to the major alleles in the MSA matrix constructed by only overlapping reads (e.g., Karect, Coral, and ECHO) [14–16].

Although the *k*-mer spectrum methods are the easiest and fastest solutions, they are unable to reliably distinguish errors from polymorphisms within repeats. Because if repeat size is larger than *k*-mer, the major *k*-mer in the spectrum may actually come from other repeat copies and lead to false correction [17, 18]. The suffix tree/array algorithms can use different sizes of *k* adaptively for reducing repeat ambiguity but they are still limited by larger repeats. Theoretically, overlap-based correction is least affected by repeats due to the usage of entire reads instead of smaller *k*-mers, and thus errors can be more reliably corrected by alleles from overlapping reads only. However, the speed of MSA is much slower owing to the complexity of computing MSA. Recently, Karect showed that that efficiency of overlap correction based on MSA can be greatly improved by representing multiple-aligned sequences as a partial-order graph [14], whereas identical sequences are collapsed into the same path and alignment can be performed for only once.

Over the past decade, FM-index is the preferred data structure underlying many state-of-the-art short-read aligners (e,g, BWA, Bowtie) [19]. These aligners have been widely used to align huge amount of short reads against reference genome with good speed and accuracy. It is mainly because FM-index compresses identical substrings into suffix array (SA) intervals, which saves both time and space for numerous repetitive sequences widespread in the genome [20]. However, to our best knowledge, FM-index was mainly used (or fine tuned) for aligning individual reads against reference genome instead of optimized for MSA, which is the most time-consuming step in overlap-based error correction.

This paper presented a novel overlap-based error correction algorithm using FM-index (called FMOE). Given a query read to be corrected, FMOE first identifies its overlapping reads by simultaneous alignment against multiple reads compressed in FM-index. Subsequently, sequencing errors are corrected by *k*-mer voting from overlapping reads only. The details of methods are described in the following section and the readers would be better familiar with either suffix tree/array, Burrows-Wheeler Transform, or FM-index.

## Methods

Our correction algorithm follows the overlap-based correction paradigm. Given a query read (to be corrected), we first identify reads overlapping with the query by performing alignment against reads compressed in FM-index, construct a MSA matrix, and replace the less-frequent alleles on the query (i.e., errors) with the most-frequent one at the same locus. The identification of overlapping reads is the most time-consuming step. This paper addresses this key issue by using the compressed feature of FM-index, which compresses identical substrings into continuous SA intervals. Our method aims to perform alignment over identical substrings only once by combining the seed-and-extend strategy with FM-index extension [21], which is described below.

**Fig. 1:**
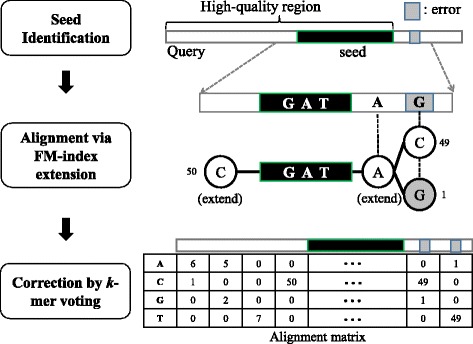
Workflow of overlap-based error correction using FM-index. Initially, a seed is identified from the high-quality regions with sufficient *k*-mer frequency. Subsequently, the flanking sequences are extended from the seed base by base using FM-index and are aligned against the query read. Note that GATA is a compression of all such strings in all reads. Finally, the most-frequent allele in each column of MSA will replace the error allele with lower frequency

Nowadays, nearly all the alignment tools (e.g., BLAST, BWA) utilize the seed-and-extend strategy for speedup, which only align reads having common seed with the query [22]. Our algorithm also used this strategy for speedup, which is divided into three steps (see Fig. 1). The first step identifies high-confident seed (i.e., *k*-mer with sufficient frequency) in the query read. Subsequently, the sequences flanking the seed (i.e., overlapping reads) are forward/backward extracted using FM-index extension, and simultaneously, each of the extended sequence is aligned against the query read. Finally, the extended sequences with sufficient identity and overlap thresholds will form a MSA matrix, and the most-frequent *k*-mer will replace the error *k*-mer with lower frequency at each erroneous locus. The details of each step are presented in the following sections.

### Seed identification

**Fig. 2:**
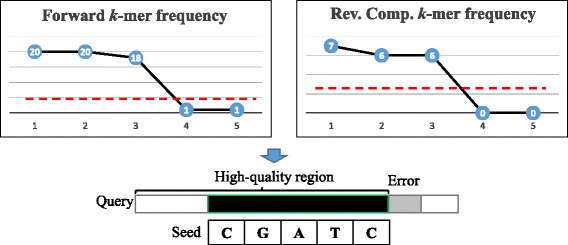
Illustration of seed identification. The high-quality region is defined as the region where *k*-mer frequencies at both forward and reverse-complement strands are above a threshold. The seed is identified as the *k*-mer closest to the error base within the high-quality region. If there are multiple high-quality regions, the largest one is selected for seed identificaiton

The forward and reverse strand sequences of inputted reads are first used to construct forward and reverse FM-indices using Li’s ropebwt2 algorithm [22]. A *k*-mer frequency spectrum (*k*=31 by default) is constructed by randomly sampling 10,000 *k*-mers from the FM-index, which can provide various statistics required at different steps (e.g., mean, median). A *k*-mer window is slided across the entire query read for detection of potential error bases, in which the *k*-mer frequencies at both strands drop below a threshold *t* (default: median *k*-mer frequency*0.5)(see Fig. 2). The region with sufficient *k*-mer frequency flanking the error base is termed high-quality region. Within this region, the *k*-mer most close to the error base is identified as seed. The identified seed will be used to extract overlapping reads for alignment later. Most reads will contain only one high-quality region at 5’ end and errors at 3’ end, owing to the limitation of Illumina sequencing by synthesis at later cycles. However, we observed few reads can be divided into multiple high-quality regions by two or more sequencing errors. The seed will be selected from the largest high-quality region in such case.

### Extraction of flanking sequences by FM-index extension

**Fig. 3:**
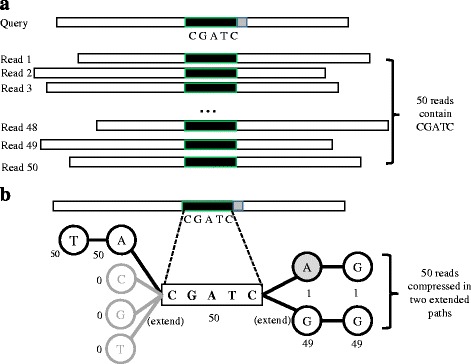
Comparison of traditional seed-and-extend alignment versus compressed alignment using FM-index extension. **a** Without compression, all reads containing the seed will be individually aligned against the query in order to compute overlap and similarity; **b** With compression, all reads containing the seed will be represented by different paths during FM-index extension. These reads will be gradually extended (extracted) from the seed sequence at both directions via FM-index extension, while identical sequences are still compressed in a single path. Simultaneously, each newly extended base will be aligned against the query read

The second step of our algorithm aims to extract and to align reads overlapping with the query using FM-index. Traditional seed-and-extend alignment algorithms independently align each read (containing the seed) against the query in order to identify overlapping reads (Fig. 3 a). Instead of independent alignment, because FM-index compressed all substrings of reads into SA intervals, it allows simultaneous alignment of multiple (compressed) substrings against the query (Fig. 3 b). Below we first describe the FM-index extension algorithm which aims to (base-by-base) extract reads (containing the seed) while compressing identical substrings as one representative path sequence. In next section, we will present a faster heuristic alignment of each path sequence against the query in order to identify mismatches and indels.

Given a seed, the flanking sequence is extended (toward both forward and backward directions) using a variant of the backward-search algorithm called FM-index extension [20, 21]. The implementation details of FM-index extension can be found in the Additional file 1, and the high-level idea is briefly described below. In the FM-index extension algorithm (Fig. 3 b), a tree data structure is used to store all the extended bases, where each tree path represents a sequence compressed by multiple identical substrings in the reads. Each tree node contains the extended base and two SA intervals (in forward/reverse FM-indices). Initially, the forward/reverse SA intervals of the seed (e.g., CGATC) are computed using the backward-search algorithm [20]. Subsequently, we recursively extend the seed sequences by querying FM-index for all possible {A,T,C,G}-extensions. This is achieved by updating the SA intervals of leaf nodes using the original algorithm of backward search for {A,T,C,G} (see Additional file 1). In addition, because forward and reverse FM-indices are built in advance, forward and backward extensions can be implemented in similar way by using FM-index of forward and reverse FM-indices.

If two or more possible {A,T,C,G}-extensions exist, new tree nodes will be created to keep all possible extension paths. Note that each path sequence represents a compression of multiple identical overlapping reads. We observed that the extension of sequences containing sequencing errors is often with lower frequency, which can be obtained according to the size of SA intervals. Thus, the extension path with insufficient frequency is pruned for speedup and for reducing search space (default: < 3) (see Additional file 1: Figure S1). The entire extension process aborts if the extended length exceeds the read length or the number of extension paths exceeds the maximum number allowed (default: 64).

### Faster heuristic alignment and similarity measurement during FM-index extension

However, many reads may occasionally contain the seed but do not actually overlap with the query (i.e., dissimilar at flanking sequences). Moreover, the precise aligned positions between query and each extended sequence are required for building an alignment matrix in the last correction stage. Consequently, we have to align each representative sequence against the query during extension in order to identify mismatches/indels and measure the similarity at the same time. Traditional dynamic-programming alignment of query read against all tree paths can give exact and accurate solution but is time-consuming. Below, we present a heuristic alignment algorithm based on commonly-shared SA intervals between the query and extended sequences, which is performed along with the extension and can tolerate both mismatches and indels.

**Fig. 4:**
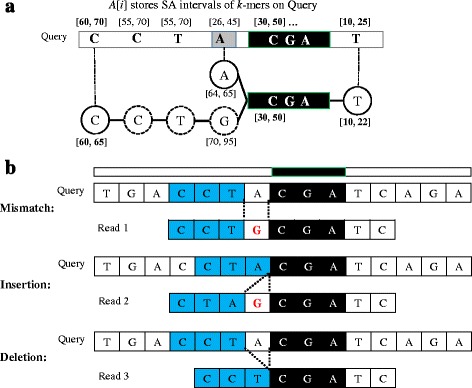
Illustration of heuristic alignment using common SA intervals during FM-index extension. **a** The SA intervals of all *k*-mers (*k*=3) in query are pre-computed and stored in an array *A*[*i*]. The forward-extended base (T) has an SA interval [10,22] inclusive within the interval [10,25] in *A*[*i*], which implies that the query and the compressed reads share common *k*-mer (GAT) at this locus; **b** Mismatches and indels will temporarily lead to no common *k*-mers (i.e., SA intervals in FM-index) found. In order to tolerate indels, the SA interval of any newly-extended base must be compared with those in a range according to the maximum indels allowed

In order to match each newly-extended interval/base against the query, we pre-compute the SA intervals of all *k*-mers in the query and store them in an array (*A*) (Fig. 4 a), where *A*[*i*] stores the SA interval of *i*-th *k*-mer on the query. For each newly-extended base, the new SA interval is compared against those stored in the array *A*. If the query read share a common *k*-mer with the newly-extended sequence (i.e., match), the SA interval of the newly-extended base will be equivalent to or inclusive within that of *A*[*i*] (e.g., the forward-extended T has a common interval [10,22] with query). On the other hand, if there are mismatches, the SA interval of extended-mismatched bases will have no common SA intervals with those in *A*. In other words, the identification of common SA intervals is similar to finding common *k*-mers between the query and representative sequence, because all substrings are compressed into SA intervals in FM-index.

Unfortunately, the presence of indel errors will shuffle the loci of common SA intervals between the query and extended sequence. As a result, the common SA intervals are not necessary at the same locus in the query and extended sequence. Figure 4 b illustrates the problem using uncompressed sequences with mismatches or indels as examples. For mismatches, the common SA intervals will be only temporarily missed and recovered at succeeding locus. For insertion or deletion errors, the common SA intervals will be found at preceding or succeeding loci in the query and extended sequence, respectively. Therefore, in order to identify common SA intervals while tolerating indels of maximum size *d*, we have to compare any new SA interval with those stored from *A*[*i*−*d*] to *A*[*i*+*d*]. Once any common SA interval is found, the aligned position between the query and extended sequence (i.e., loci of the common SA intervals) can be thus known and recorded, which will be used for building an alignment matrix in the last correction step.

Furthermore, because any mismatch or indel will reduce this number of common SA intervals, the similarity between the query and each extended sequence can now be computed by the number of common SA intervals divided by total number of SA intervals. The extended sequence with low similarity with the query can be thus immediately discarded without further extension. As Illumina platform has typical error rate around 1%, we discard extended sequences with error rate below 5% by default. This pruning-by-similarity procedure during FM-index extension also significantly improves the speed of our algorithm.

The proposed alignment algorithm is heuristic speedup in comparison with exact alignment algorithm using dynamic programming. We compare the time complexity of our alignment with that of exact algorithm in order to identify the source of speedup. Note that each extension is an update of existing SA interval which takes only *O*(1) time. Therefore, the FM-index extension takes *O*(*r* *w*) time, where *r* is the read length and *w* is the maximum tree paths allowed. Because we need to tolerate maximum *d* indels by sequential search a range of SA intervals, the entire algorithm takes *O*(*r* *w* *d*) time. Theoretically, the sequential search of SA intervals can be replaced by binary search using interval tree, which leads to better *O*(*r* *w* log*d*) time. But practically, we didn’t gain better efficiency mainly because *d* (indel) is very small in Illumina platforms. In comparison with independent alignment approach, which takes *O*(*r* *n* *d*) time where *n* is the total number of reads if using *d*-banded dynamic programming speedup, our method is still much faster as *w* is much smaller than *n*. When tested on real datasets, we further observed that, during FM-index extension, only one or two paths are extended for most reads instead of worst-case *O*(*w*) paths. This is probably due to the high sequencing accuracy of Illumina platforms. Therefore, the algorithm can run in almost linear time in practice.

### Correction via *k*-mer voting from overlapping reads

**Fig. 5:**
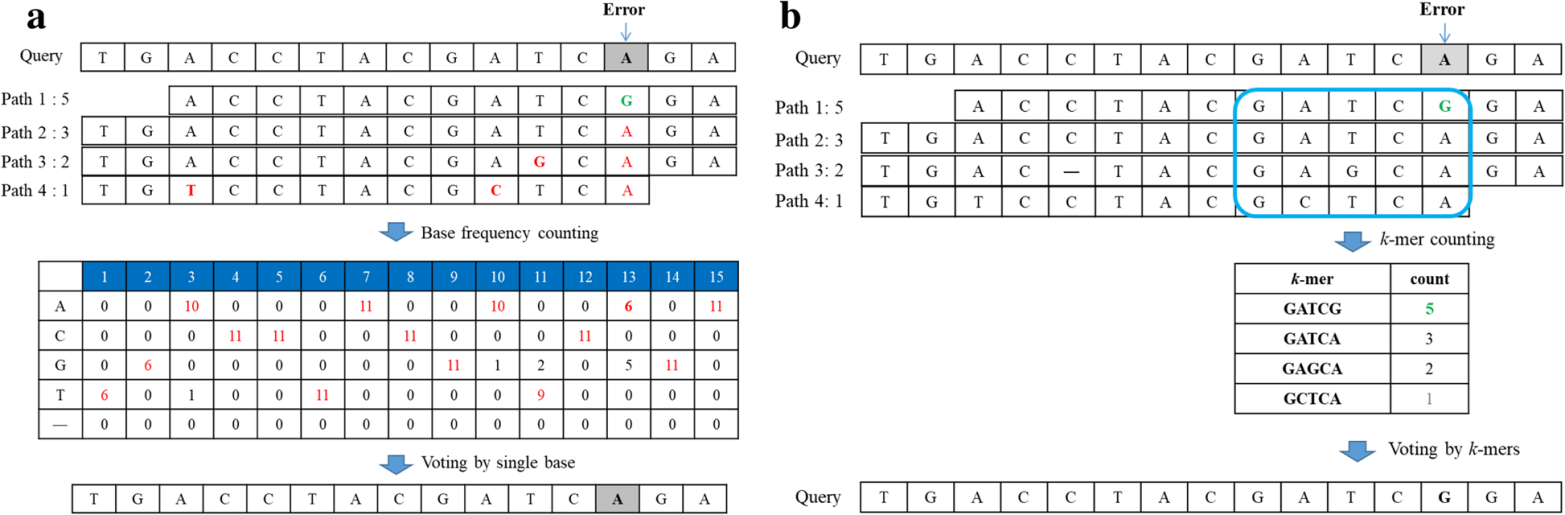
Comparison of single-base voting versus *k*-mer voting versus. **a** The alignment matrix of reads overlapping with query is constructed. In single-base voting, the most-frequent allele at each column will replace the less-frequent one on query. However. the major allele is not necessary the correct base in error-prone region; **b** In *k*=mer voting, the *k*-mer frequency over the error base is derived from previous FM-index extension. The *k*-mer with highest frequency at the locus will be chosen to replace the original allele. This is particularly useful in high-GC regions where the most frequent allele may not be accurate

Existing overlap-correction methods often build a MSA matrix after alignment of overlapping reads onto the query (Fig. 5 a). Based on the MSA matrix, the minor allele (i.e., error) on the query will be replaced with the most frequent allele at the same locus in the MSA matrix. However, we observed that, in high-GC regions where error rate is significantly elevated, the majority vote using single base is less accurate, because the most frequent allele at one locus can even come from errors. Nevertheless, although the error rate in these error-prone regions is high, there are still a few reads containing a short run of correct bases. As a consequence, the frequency of correct *k*-mers is larger than that of erroneous *k*-mers (default: *k*≈5). Therefore, the error correction is done by *k*-mer voting instead of voting by a single base. Specifically, we first count the *k*-mer frequency over the error based on overlapping reads derived from previous stage. Because identical reads are compressed into one extension path represented by a SA interval, the frequency of each *k*-mer in these overlapping reads can be known easily according to the size of SA interval. The most frequent *k*-mer in the MSA matrix will be used to replace the erroneous *k*-mer on the query (Fig. 5 b). Note that this correction approach only compute the *k*-mer frequency from overlapping reads for voting. In comparison with *k*-mer correction methods voting by *k*-mer counts from all reads, our method is more robust to repeats.

## Results

FMOE is implemented in C++ and freely available on Github (https://github.com/ythuang0522/FMOC). We compare FMOE with two other leading overlap-based methods, Karect and Coral, and four *k*-mer spectrum methods (QuorUm, RACER, BLESS2, and SGA). Table 1 lists the sequencing statistics of four test datasets with genome sizes ranging from 4.6 Mb to 100.2 Mb and various read lengths, where reads and reference genomes are downloaded from the GAGE-b project and NCBI [23]. We first compared these programs in terms of correction power and accuracy. The correction power and accuracy are evaluated by BWA-aligning reads onto the corresponding reference genomes in order to compute the aligned length, identity, and indels. Subsequently, we move to investigate the influence of different correction algorithms on the genome assembly.

**Table 1:** Genome and sequencing statistic of four data sets used in the experiments

	Genome size	Num. reads	Read length	Coverage
M. abscessus	5.09M	2.03M	251 bp	100x
R. sphaeroides	4.6M	1.75M	251 bp	95x
E. coli	4.6M	1.69M	150 bp	54x
C. elegans	100.2M	68.27M	110 bp	74x

### Correction power and accuracy

Tables 2, 3, 4 and 5 list the correction power (in terms of total corrected bases and corrected read length) and accuracy (in terms of identity and indel rate) of each tool across four datasets (*M*.*a* *b* *s* *c* *e* *s* *s* *u* *s*, *R*.*s* *p* *h* *a* *e* *r* *o* *i* *d* *e* *s*, *E*.*c* *o* *l* *i*, and *C*.*e* *l* *e* *g* *a* *n* *s*). In general, the results indicated the FMOE obtains the best correction power in comparison with all other overlap-based or *k*-mer based methods in long-read datasets. Overlap-based methods tend to have higher correction power mainly due to the ability of correcting error-prone reads in high-GC regions using time-consuming alignment. On the other hand, *k*-mer based methods usually have high accuracy and runs much faster owing to the skip of error-prone regions. QuorUm obtains the best accuracy because it sacrifices the correction power by trimming or throwing away uncorrected reads. When restricted to overlap-based methods, the accuracy of FMOE (in terms of identity and indel rate) is best than the others in most datasets. The identify of Karect is slightly better than FMOE in the *E*.*c* *o* *l* *i* and *C*.*e* *l* *e* *g* *a* *n* *s* datasets, but its indel rate is worse than that of FMOE.

**Table 2:** Correction power/sensitivity (in terms of total corrected bases and corrected read length) and accuracy (in terms of identity and indel rate) of seven methods over M. abscessus dataset (5 Mb genome, 251 bp read length)

	Corrected bases (bp)	Corrected length (bp)	Identity (%)	Indel rate (10^−5^)
Raw data	353.43 Mbp	180.77	98.80%	4.37
Coral	358.65 Mbp	182.72	99.19%	4.59
Karect	387.17 Mbp	196.62	99.42%	2.91
FMOE	492.95 Mbp	249.31	99.85%	1.37
QuorUm	361.02 Mbp	182.55	99.96%	0.56
RACER	381.11 Mbp	192.49	99.89%	1.23
BLESS2	358.02 Mbp	182.35	99.87%	0.68
SGA	366.05 Mbp	182.16	99.46%	2.35

Coral, Karect, and our FMOE are overlap-based correction methods whereas QuorUm, RACER, BLESS2, and SGA belong to *k*-mer spectrum approach

**Table 3:** Correction power and accuracy of seven methods over R. sphaeroides dataset (4.6Mb genome, 251bp read length)

	Corrected bases (bp)	Corrected length (bp)	Identity (%)	Indel rate (10^−5^)
Raw data	286 Mbp	183.08	96.07%	11.16
Coral	309.76 Mbp	195.41	97.47%	7.02
Karect	359.93 Mbp	223.01	98.90%	1.80
FMOE	393.06 Mbp	243.11	99.41%	1.55
QuorUm	299.23 Mbp	185.81	99.96%	0.17
RACER	332.67 Mbp	204.78	99.74%	1.23
BLESS2	293.95 Mbp	185.55	99.53%	0.8
SGA	300.83 Mbp	187.98	98.20%	9.19

**Table 4:** Correction power and accuracy of seven methods over E. coli dataset (4.6 Mb genome, 150 bp read length)

	Corrected bases (bp)	Corrected length (bp)	Identity (%)	Indel rate (10^−5^)
Raw data	229.94 Mbp	144.13	99.22%	1.90
Coral	231.09 Mbp	144.07	99.75%	1.37
Karect	236.90 Mbp	146.86	99.92%	0.76
FMOE	239.99 Mbp	149.46	99.85%	0.45
QuorUm	231.20 Mbp	143.77	99.97%	0.12
RACER	234.89 Mbp	145.99	99.94%	0.73
BLESS2	234.42 Mbp	143.75	99.92%	0.28
SGA	232.26 Mbp	144.14	99.81%	1.98

**Table 5:** Correction power and accuracy of seven methods over C.elegans dataset (100.2Mb genome, 110bp read length)

	Corrected bases (bp)	Corrected length (bp)	Identity (%)	Indel rate (10^−5^)
Raw data	7376.84 Mbp	109.32	99.54%	14.91
Coral	7389.51 Mbp	109.34	90.70%	15.47
Karect	7041.49 Mbp	109.49	99.79%	13.93
FMOE	7411.06 Mbp	109.57	99.78%	12.45
QuorUm	7402.77 Mbp	109.24	99.8%	11.64
RACER	7429.30 Mbp	109.61	99.85%	10.69
BLESS2	7407.09 Mbp	109.44	99.86%	8.9
SGA	7420.71 Mbp	109.58	99.83%	11.71

Theoretically, the overlap-based methods gain more benefits when processing longer reads in comparison with shorter reads. By comparing the long-read and short-read datasets (i.e., 251 bp, 150 bp, and 110 bp in Tables 2, 3, 4 and 5), we observed that our method FMOE has larger power than the others in the long-read datasets (e.g., 251 bp datasets). For instance, FMOE outputted average 249 bp-corrected reads whereas the 2nd best Karect only produced 196 bp-corrected reads in the *M*.*a* *b* *s* *c* *e* *s* *s* *u* *s* dataset with 251 bp reads. On the other hand, in the short-read dataset (e.g., 150 bp and 110 bp in Tables 3 and 4), the benefits of using overlap-based methods are diminishing and thus the results are not much different from those of *k*-mer based method.

In terms of speed, the *k*-mer based methods are all faster than overlap-based methods due to the lack of alignment and sacrificed power (Table 6). We observed the speed of FMOE and Karect are almost the same and both run much faster than Coral. Karect is based on partial-order alignment (POA) graph, where the identical aligned bases are collapsed into a single vertex in the graph. This implies that the compressed extension using FM-index within FMOE is similar to the POA graph, because identical sequences are also compressed into the same path sequences during FM-index extension.

**Table 6:** Comparison of running time

	M. abscessus	R. sphaeroides	E. coli	C. elegans
Coral	03:48:32	02:03:19	00:52:45	43:33:50
Karect	00:14:41	00:30:46	00:03:07	01:41:15
FMOE	00:14:51	00:14:22	00:03:51	02:02:32
QuorUm	00:01:28	00:03:50	00:00:27	00:23:54
RACER	00:03:14	00:02:58	00:00:54	00:19.28
BLESS2	00:03:02	00:01:33	00:00:34	00:19:18
SGA	00:05:51	00:23:46	00:02:48	00:43:58

### Comparison of assembly results

The reads corrected by different methods are further tested for genome assembly in order to understand the influence of correction to assembly. Existing genome assemblers are further classified into de Bruijn graph and overlap graph assemblers, which are suitable for assembling short and long reads. In order to reduce the influence from different assembly graph models, these corrected reads are assembled using a hybrid-graph assembler called StriDe which captures the features of of both de Bruijn and overlap graphs [21]. Nevertheless, we would like to note that optimized selection of genome assemblers and parameter tuning for each correction algorithm may change the results provided below. Consequently, the following results are generated by the default parameters of StriDe assembler, which only serves as one reference of possible influence of each correction algorithm to assembly. The complete evaluation of assembly is beyond the scope of this paper which focuses on error correction.

Tables 7, 8, 9 and 10 list the number of contigs, N50, NA50, misassemby, and sum of assembled bases of the seven correction algorithms tested over the four datasets. All the assembly metrics are computed by QUAST [24]. In the *M*.*a* *b* *s* *c* *e* *s* *s* *u* *s* dataset (Table 7), FMOE obtains the best assembly contiguity and accuracy as a whole (according to NA50). Karect, FMOE, QuorUm, RACER, and BLESS2 nearly assembled the expected 5 Mbp genome size, whereas Coral and SGA are significantly worse than the others in terms of contiguity (N50), accuracy (NA50 and misassembly), and completeness (Sum). In the *R*.*s* *h* *p* *a* *e* *r* *i* *o* *i* *d* *e* *s* dataset (Table 8), QuorUm outperforms the others in general. Further investigation indicates that this dataset is of very low sequencing quality compared with the others. The trimming procedure in QuorUm may be better and suitable for the low-quality datasets. In terms of assembly completeness, Coral only assembled a partial genome, whereas the genome size assembled by FMOE is most close to the expected 4.6 Mbp. This implies the optimization for correction power can help assembly completeness in low-quality sequencing. In the *E*.*c* *o* *l* *i* dataset, QuorUm and BLESS2 outperforms the others in terms of NA50, and assembly completeness of FMOE is slightly better. In large genome dataset (*C*.*e* *l* *e* *g* *a* *n* *s*), QuorUm performs much worse than all the overlap-based methods in most metrics. FMOE, RACER, and BLESS2 perform similarly and better than the others. When restricted to overlap-based correction methods, FMOE and Karect performs almost the same in short-read datasets (e.g., *C*.*e* *l* *e* *g* *a* *n* *s*), and FMOE is much more sensitive than the others in long-read datasets (e.g., *M*.*a* *b* *s* *c* *e* *s* *s* *u* *s* and *R*.*s* *p* *h* *a* *e* *r* *o* *i* *d* *e* *s*).

**Table 7:** Assembly results of M. abscessus

	No. Ctg.	N50	NA50	Misassemblies	Sum
Coral	1,053	6,166	4,216	628	4,505,117
Karect	201	43,116	34,962	42	5,081,371
FMOE	75	144,110	120,739	7	5,138,819
QuorUm	66	138,549	116,520	5	5,139,557
RACER	294	27,986	26,936	16	5,105,201
BLESS2	139	70,560	64,289	34	5,073,140
SGA	41	885	437	55	36,626

**Table 8:** Assembly results of R. sphaeroides

	No. Ctg.	N50	NA50	Misassemblies	Sum
Coral	1,196	4,010	3,911	34	3,497,317
Karect	464	15,750	15,723	17	4,394,367
FMOE	225	73,476	71,472	1	4,558,663
QuorUm	123	127,719	127,659	4	4,528,808
RACER	921	7,126	6,982	17	4,126,662
BLESS2	833	6,573	6,549	11	3,522,646
SGA	952	6,372	6,172	51	3,965,387

**Table 9:** Assembly results of E. coli

	No. Ctg.	N50	NA50	Misassemblies	Sum
Coral	257	95,730	95,483	4	4,560,741
Karect	392	107,932	105,790	1	4,565,614
FMOE	375	112,502	112,502	1	4,628,706
QuorUm	211	132,749	132,749	4	4,564,093
RACER	84	107,449	95,962	10	4,545,995
BLESS2	94	133,195	133,195	1	4,617,496
SGA	402	90,858	90,858	1	4,628,475

**Table 10:** Assembly results of C.elegans

	No. Ctg.	N50	NA50	Misassemblies	Sum
Coral	12,846	16,343	15,265	868	90.27 Mbp
Karect	13,252	18,984	17,922	429	90.56 Mbp
FMOE	14,243	17,676	16,768	422	90.27 Mbp
QuorUm	18,486	11,491	11,461	36	89.89 Mbp
RACER	14,233	17,442	16,458	638	90.26 Mbp
BLESS2	14,246	17,047	16,533	591	90.66 Mbp
SGA	23	743	390	4	18,304 bp

## Discussion and conclusion

This paper presented a novel overlap-based correction algorithm for NGS reads using FM-index. Our results indicated our method has larger correction power (in terms of corrected bases and read lengths) at comparable accuracy in comparison with others. We observed the compressed feature of FM-index runs almost at the same speed as a POA approach called Karect, This implies that both the partial-order graph used by Karect and FM-index used by our algorithm compressed identical reads in different manners in order to accelerate the multiple sequence alignment. In addition, we observed that, in the low-quality dataset, the *k*-mer based methods are superior in terms of assembly. This indicates that extremely low-quality reads may be better trimmed or discarded instead of trying to correct them for maximizing the correction power. Consequently, better correction algorithms for genome assembly may be achieved by striking a balance between correction power and accuracy for high- and low-quality reads.

## Additional file

Additional file 1 Supplementary method and figures are provided in this file. (PDF 661 kb)
