# Supplementary material for: An efficient error correction algorithm using FM-index
Source: BMC Bioinformatics. 2017 Nov 28;18:524. doi: 10.1186/s12859-017-1940-1 (PMC5704532; doi:10.1186/s12859-017-1940-1)
Supplement: Additional file 1 — Supplementary method and figures are provided in this file. (PDF 661 kb) [file 12859_2017_1940_MOESM1_ESM.pdf]

An Efficient Error Correction Algorithm Using FM-index

(Supplementary Material)

Yao-Ting Huang and Yu-Wen Huang
Department of Computer Science and Information Engineering, National Chung Cheng University, Chiayi, Taiwan.

Supplementary Methods

The FM-index is a substring index based on Burrows-Wheeler Transform (BWT). We chose Li's ropebwt2 algorithm as it is able to index ten millions of reads within a few minutes and billions of reads within several hours. We ported the original C programs into our C++ implementation. In order to address the uncertainty of strandness of each read and to provide both forward/backward extension, two FM-indices representing the forward and reverse sequences are constructed. The constructed FM-indices are further compressed using Run-Length Encoding (RLE) for reducing disk/memory usage, and the random access of RLE-compressed BWT is provided by the two-level indexing in SGA (Simpson and Durbin, 2012).

In the FM-index, all the substrings are compressed as Suffix Array (SA) intervals. The extension of any substring *S* can be achieved by directly updating the SA interval of *S* using the backward-search algorithm with {A,T,C,G}. Specifically, let $C[c]$ be a table stores the number of occurrences of characters lexically smaller than *c*. Define $Occ[c,k]$ as the number of occurrences of character *c* in the prefix of BWT[1..*k*]. $C[c]$, $Occ[c,k]$, and BWT are collectively termed FM-index. The extension of any SA interval ([*first*,*last*]) is computed by running a variant of backward-search algorithm shown below:

```
while (Length < Read_Length && NumLeaf < Max_Leafs)
{
```

$$\begin{matrix} first = C[c] + Occ[c, first - 1] \\ last = C[c] + Occ[c, last] - 1 \end{matrix}, c = \{A, C, G, T\}.$$

```
}
```

The above FM-index extension may produce zero to maximum four extension paths at each iteration. However, not every extended path will be retained. Unless the frequency of the new path (i.e., SA interval size) is sufficient (min. 3), the extension path will not be kept for further extension. In addition, the implicit substring (overlap)

increases with respect to each update. The advantage of using FM-index extension is the ability to extend through repeats larger than the initial k-mer. The drawback is the implicit overlap will eventually exceed maximum read length. Therefore, whenever the extension of SA intervals failed, a new suffix of length k must be extracted from the extended sequence, re-compute the SA interval of new suffix, and try extension by updating the new interval. Nevertheless, this extra cost can still be amortized into majority amount of $O(1)$ successful extensions.

Supplementary Figures

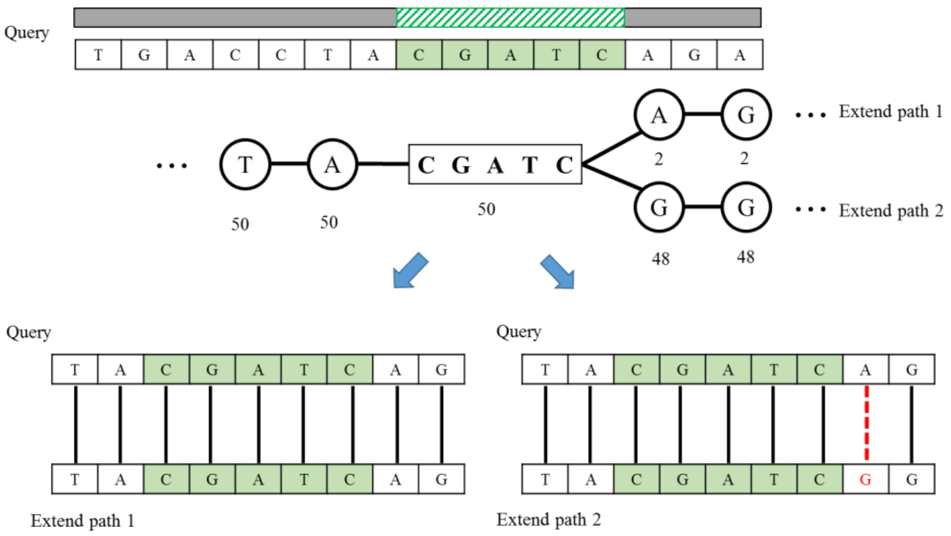

Supplementary Figure S1: Illustration of two extended paths where the top-right path contains errors and the down-right path is the error-free path. Because the size of newly extended SA interval represents the frequency, the top-right path can be pruned immediately once the frequency drops below a threshold (5 by default). This pruning procedure can boost the efficiency of FM-index extension.
